# Early Thoracic Duct Ligation for Post-esophagectomy Chyle Leak: A 10-Year Institutional Experience With Early Operative Intervention

**DOI:** 10.7759/cureus.103318

**Published:** 2026-02-09

**Authors:** Parvinder Singh, Gurmeet Singh, Gurlal Singh, Pawan Kumar, Gaurav Goel

**Affiliations:** 1 Surgical Oncology, Advanced Cancer Institute, Baba Farid University of Health Sciences (BFUHS), Bathinda, IND; 2 Preventive Oncology, Advanced Cancer Institute, Baba Farid University of Health Sciences (BFUHS), Bathinda, IND; 3 Surgical Gastroenterology, Advanced Cancer Institute, Baba Farid University of Health Sciences (BFUHS), Bathinda, IND; 4 Radiation Oncology, Advanced Cancer Institute, Baba Farid University of Health Sciences (BFUHS), Bathinda, IND

**Keywords:** chylothorax, complications, conservative management, esophagectomy, high-output chylothorax

## Abstract

Background and aim

Chyle leak is a rare but potentially lethal complication following esophagectomy. Optimal timing for thoracic duct ligation remains unclear, particularly for high-output leaks. This study aimed to evaluate outcomes of early thoracic duct ligation compared with conservative therapy in post-esophagectomy chyle leak.

Methods

This is a retrospective study of 500 patients undergoing esophagectomy from 2016 to 2025 at a tertiary cancer center. Six patients (1.2%) developed chyle leak. Diagnosis was based on high-volume drainage, milky effluent after feeding, and biochemical confirmation. Conservative treatment included drainage, nil per oral, and total parenteral nutrition. Early thoracic duct ligation was performed via a transthoracic or transabdominal approach. Statistical tests included the t-test and Fisher’s exact test.

Results

Patients with chyle leak were significantly younger than those without leak (44.0±5.7 vs. 53.2±13.4 years; p=0.009). Conservative therapy (n=1) resulted in persistent leak and death (100% mortality). Early surgical ligation (n=5) achieved 100% resolution with no mortality and shorter hospital stay (13-14 days vs. 18 days). Overall mortality in the chyle-leak group was 16.7%, exclusively in the conservatively treated patient.

Conclusion

Early thoracic duct ligation appears to be a safe and effective strategy in selected high-output post-esophagectomy chyle leaks in our institutional experience. Prolonged conservative therapy in high-output leaks is associated with avoidable morbidity and mortality.

## Introduction

Chyle leak following esophagectomy, although uncommon, is associated with significant morbidity. Reported incidence ranges from 1% to 4% in major surgical series [1-3]. Loss of lymphatic fluid leads to progressive depletion of proteins, fats, electrolytes, and lymphocytes, resulting in immunosuppression, malnutrition, respiratory compromise, and increased susceptibility to infection [4-6]. Historically, mortality from untreated or delayed chyle leak was substantial [5,6].

Mechanisms contributing to thoracic duct injury include extensive mediastinal lymphadenectomy, difficult dissection in the middle third of the esophagus, and unrecognized variations in thoracic duct anatomy [7,8]. Such anomalies complicate intraoperative identification. Early diagnosis is crucial but often delayed due to non-specific post-operative symptoms such as dyspnea or increased drain output.

Conservative management, nil per oral, total parenteral nutrition, and continued drainage are generally recommended for low-output leaks. However, high-output leaks seldom resolve without surgery and are associated with metabolic deterioration and increased mortality [3,9]. Surgical interventions described in literature range from thoracotomy to minimally invasive thoracic duct ligation, with excellent outcomes when performed early [7,10,11].

Additional diagnostic and therapeutic adjuncts, such as lymphangiography and thoracic duct embolization, have gained attention in recent years, though accessibility varies significantly across institutions [12-15]. Prophylactic thoracic duct ligation remains controversial, with mixed outcomes reported [16]. Given these uncertainties, institutional experiences contribute valuable insight. This study analyzes a decade of post-operative outcomes at a tertiary cancer center, focusing on the role of early surgical ligation compared with conservative management.

## Materials and methods

This retrospective, descriptive, observational study was conducted at the Advanced Cancer Institute, Bathinda, and included all patients who underwent esophagectomy between January 2016 and October 2025. Both transthoracic (Ivor Lewis) and transhiatal esophagectomies were included. Patient demographics, operative variables, post-operative course, and outcomes were collected from institutional records.

For the purpose of this study, high-output chyle leak was defined as drainage ≥800 mL/day persisting for more than 48 h; milky drainage following the initiation of enteral feeding; or biochemical confirmation with pleural fluid triglyceride level exceeding 110 mg/dL or detection of chylomicrons. Once diagnosed, all patients were placed on a standardized management pathway.

Following confirmation of post-operative chyle leak, all patients were evaluated for daily drain output, clinical stability, and biochemical parameters. Patients and their attendants were formally counselled regarding the available treatment options, including conservative management and early thoracic duct ligation. The potential benefits, risks, likelihood of success, and possible complications of each approach were explained in detail.

While objective clinical criteria, such as drainage volume, persistence of leak, and physiological deterioration, guided the treating team’s recommendation, the final management decision was made through a shared decision-making process after obtaining informed consent from the patient or attendant. Conservative management was considered for low-output leaks. Early thoracic duct ligation was advised for high-output leaks (≥800 mL/day) based on institutional practice favoring definitive control. Final treatment decisions were individualized after shared clinical counseling.

Conservative therapy consisted of continued drainage, nil per oral status, total parenteral nutrition, intravenous fluids, and electrolyte correction. Octreotide was used selectively based on clinical judgment and drainage pattern. Early thoracic duct ligation was defined as operative intervention within 48-72 h of confirmed diagnosis. All surgeries were performed by experienced thoracic and gastrointestinal oncology surgeons via either a right thoracotomy or a transabdominal suprahiatal approach. Fatty cream (100-200 mL) was administered via jejunostomy 2 h prior to the procedure to aid intraoperative identification of the thoracic duct.

The primary endpoint was resolution of chyle leak, defined as a reduction in chest drainage to less than 200 mL/day of non-milky fluid. Secondary endpoints included length of hospital stay, post-operative complications, and mortality. Statistical analysis was performed using SPSS Statistics version 26.0 (Armonk, NY: IBM Corp.) for Windows. Continuous variables were compared using Student’s t-test and categorical variables using Fisher’s exact test. Statistical significance was set at p<0.05.

This study was approved by the Institutional Ethics Committee of Guru Gobind Singh Medical College, Faridkot (#GGS/IEC/014, dated June 17, 2025), and all information was collected in accordance with the ethical standards of the ICMR National Ethical Guidelines for Biomedical and Health Research involving human participants.

## Results

A total of 500 patients underwent esophagectomy during the study period, of whom six (1.2%) developed post-operative chyle leak. Baseline characteristics for all patients are summarized in Table 1. Patients with chyle leak were significantly younger compared with those without leak (44.0±5.7 vs. 53.2±13.4 years; p=0.009). No statistically significant differences were observed between groups in sex distribution, operative duration, blood loss, or mean hospital stay (Table 2).

**Table 1:** Baseline characteristics of all esophagectomy patients (n=500). GE: gastroesophageal

Parameters		Values
Age, years (mean±SD)		53.2±13.4
Sex, n (%)	Male	230 (46)
	Female	270 (54)
Histopathology, n (%)	Squamous cell carcinoma	325 (65)
	Adenocarcinoma	175 (35)
Tumor location, n (%)	Upper third	90 (18)
	Middle third	235 (47)
	Lower third/GE junction	175 (35)
Surgical approach, n (%)	Transthoracic	350 (70)
	Transhiatal	150 (30)
Duration of surgery, hours (mean±SD)		5.6±1.1
Blood loss, mL (mean±SD)		1020±380
Mean hospital stay, days (mean±SD)		14.3±4.9
ICU stay, days (mean±SD)		3.2±1.4

**Table 2:** Comparison of variables between patients with and without chyle leak. *Student’s t-test for continuous variables and Fisher’s exact test or chi-square test for categorical variables. **P<0.05 was statistically significant.

Parameters	Chyle leak (n=6)	No chyle leak (n=494)	Test statistic*	p-Value
Mean age, years (mean±SD)	44.0±5.7	53.2±13.4	t=2.63	0.009**
Male sex, n (%)	5 (83)	225 (46)	χ²=1.78	0.18
Duration of surgery (h)	5.8±0.9	5.6±1.1	t=0.47	0.64
Blood loss (mL)	1100±450	1020±380	t=0.60	0.55
ICU stay (days)	3.8±1.3	3.2±1.4	t=0.85	0.40
Mortality, n (%)	1 (16.7)	24 (4.8)	Fisher’s exact	0.28

Of the six patients with chyle leak, one underwent conservative management, which failed, resulting in death on post-operative day 18. The remaining five patients underwent early thoracic duct ligation, three via a right thoracotomy and two via a transabdominal approach. All five surgically treated patients demonstrated rapid cessation of chyle output, normalization of drainage, earlier drain removal, and shorter hospitalization, averaging 13-14 days (Table 3). No deaths occurred in the surgical group. Overall mortality in the chyle-leak subgroup was 16.7%, attributable solely to the patient who failed conservative therapy. Surgical ligation resulted in 100% success in 5/6 cases; the only mortality occurred in the conservatively managed patient.

**Table 3:** Management and outcomes of patients with chyle leak (n=6). POD: post-operative day

Case number	Age (years)	Sex	Output (mL/day)	Management	Surgical approach	POD of surgery	Outcome
1	42	Male	1200	Surgical ligation	Right thoracotomy	POD 3	Recovered
2	46	Male	1500	Surgical ligation	Right thoracotomy	POD 2	Recovered
3	39	Male	1000	Surgical ligation	Transabdominal suprahiatal	POD 3	Recovered
4	45	Female	900	Surgical ligation	Transabdominal suprahiatal	POD 2	Recovered
5	48	Male	1100	Surgical ligation	Right thoracotomy	POD 2	Recovered
6	44	Male	1300	Conservative (failed)	-	-	Death (POD 18)

## Discussion

Chyle leak after esophagectomy, although infrequent, represents one of the most physiologically exhausting post-operative complications. The incidence in our study (1.2%) lies within the range reported by major series, where rates between 1% and 4% have been consistently documented [1,2]. Loss of chyle results in rapid depletion of lymphocytes, proteins, fats, and electrolytes, leading to immunosuppression, malnutrition, respiratory compromise, and sepsis if inadequately addressed [5]. These metabolic and immune consequences historically contributed to high mortality when early diagnosis and treatment were not pursued [3,6].

Thoracic duct injury most commonly occurs during mediastinal lymphadenectomy and esophageal mobilization, especially in operations involving the middle third of the esophagus or in the presence of ductal anatomical variations [7,8]. Rao et al. demonstrated in their 20-year experience that thoracic duct injury during esophagectomy often results from this anatomical vulnerability and can be difficult to recognize intraoperatively [8]. Such factors complicate prevention and highlight the importance of early post-operative identification.

The role of conservative therapy in managing chyle leak remains a subject of debate. Although bowel rest, total parenteral nutrition, and adequate drainage are generally recommended for low-output leaks, numerous studies, including large esophagectomy cohorts, indicate that high-output chylothorax rarely resolves without operative intervention [3,9]. Persistent drainage leads to continued metabolic decline and is associated with increased morbidity and mortality. This was reflected in our own experience as follows: the only patient treated conservatively experienced progressive deterioration and ultimately died.

In contrast, early surgical ligation has consistently demonstrated excellent results. Brinkmann et al. reported 100% success when surgical intervention was performed promptly after diagnosis, emphasizing that delayed surgery increases complications and prolongs hospitalization [3]. Minimally invasive thoracic duct ligation techniques have further improved outcomes and reduced morbidity, as demonstrated in recent series by Gheorghe et al. [7]. Similarly, transabdominal ligation has been shown to be an effective and safe alternative approach for post-operative chylothorax, with Schumacher et al. reporting excellent long-term outcomes and minimal complications [12].

Lymphangiography can both diagnose and therapeutically reduce leak output by embolizing leaking lymphatic channels [10,11,15]. While these techniques are increasingly used in specialized centers, their availability remains limited, particularly in low- and middle-income settings. Therefore, surgery continues to be the most universally reliable intervention.

The controversy surrounding prophylactic thoracic duct ligation also warrants consideration. Although proposed as a preventive strategy, evidence remains inconclusive. Some studies suggest potential benefits, whereas others, including an updated review by Liu et al., note no clear reduction in post-operative chyle leaks and highlight variations in surgical practice and anatomical differences [16]. Given the lack of consensus, routine prophylactic ligation remains debated.

Our findings align strongly with the global literature as follows: early thoracic duct ligation is safe, effective, and associated with rapid resolution and zero mortality in high-output leaks. In contrast, conservative therapy carries a substantial risk of failure and clinical deterioration in such patients. Based on these data and existing evidence, our findings support consideration of early operative intervention in selected high-output cases, preferably within 48-72 h after esophagectomy.

Limitations

This study has several limitations that should be acknowledged. First, its retrospective design introduces the potential for selection bias and unmeasured confounding variables inherent to observational research. Second, the number of patients with post-operative chyle leak was small, reflecting the rarity of this complication and limiting statistical power for meaningful subgroup comparisons. Third, treatment allocation was not randomized and was based on clinical judgment in a real-world practice setting, which may influence the interpretation of outcomes. Fourth, advanced interventional radiologic techniques, such as thoracic duct embolization, were not routinely available at our institution during the study period, potentially limiting available treatment options. Finally, the extremely small sample size limits external generalizability, and the findings should therefore be interpreted as descriptive institutional experience rather than definitive comparative evidence. Larger multicenter studies would be required to establish stronger treatment recommendations.

## Conclusions

Early thoracic duct ligation appears to be a safe and effective treatment option for high-output post-esophagectomy chyle leak in our institutional experience. Conservative therapy may remain appropriate for selected low-output cases. Given the rarity of this complication, our findings should be interpreted as descriptive observational data that align with existing literature rather than definitive comparative evidence.

## References

[1] Lam KH, Lim ST, Wong J, Ong GB (1979). Chylothorax following resection of the oesophagus. Br J Surg.

[2] Rindani R, Martin CJ, Cox MR (1999). Transhiatal versus Ivor-Lewis oesophagectomy: is there a difference?. Aust N Z J Surg.

[3] Brinkmann S, Schroeder W, Junggeburth K, Gutschow CA, Bludau M, Hoelscher AH, Leers JM (2016). Incidence and management of chylothorax after Ivor Lewis esophagectomy for cancer of the esophagus. J Thorac Cardiovasc Surg.

[4] Ahn S, Lee H, Kang JK (2023). Intractable chylous leak after radical esophagectomy treated with radiotherapy. J Cardiothorac Surg.

[5] Valentine VG, Raffin TA (1992). The management of chylothorax. Chest.

[6] Lampson RS (1948). Traumatic chylothorax; a review of the literature and report of a case treated by mediastinal ligation of the thoracic duct. J Thorac Surg.

[7] Gheorghe M, Achim F, Hoara P, Constantin A, Constantinoiu S (2022). Management of chylothorax in esophageal surgery by minimally invasive thoracoscopic approach: case series. Chirurgia (Bucur).

[8] Rao DV, Chava SP, Sahni P, Chattopadhyay TK (2004). Thoracic duct injury during esophagectomy: 20 years experience at a tertiary care center in a developing country. Dis Esophagus.

[9] Wang S, Jiang W (2022). Post-esophagectomy chylothorax refractory to mass ligation of thoracic duct above diaphragm: a case report. J Cardiothorac Surg.

[10] Kuroda A, Yajima S, Urabe M, Yoshimura S, Ri M, Yagi K, Seto Y (2025). Post-esophagectomy chylothorax with thoracic duct anomaly successfully treated with lymphangiography: a case report. Surg Case Rep.

[11] Sato Y, Tanaka Y, Imai T (2021). Chylothorax after esophagectomy treated with inguinal intranodal lymphangiography and transvenous retrograde thoracic duct embolization. Clin J Gastroenterol.

[12] Schumacher G, Weidemann H, Langrehr JM (2007). Transabdominal ligation of the thoracic duct as treatment of choice for postoperative chylothorax after esophagectomy. Dis Esophagus.

[13] Barnes TG, MacGregor T, Sgromo B, Maynard ND, Gillies RS (2022). Near infra-red fluorescence identification of the thoracic duct to prevent chyle leaks during oesophagectomy. Surg Endosc.

[14] Franceschilli M, Argirò R, Siragusa L, Usai V, Sibio S, Di Carlo S (2022). Thoracic duct embolization for delayed chyle leak after Lewis-Tanner esophagectomy. Am J Case Rep.

[15] Shimakawa T, Naritaka Y, Miyazawa M (2017). Lymphangiography was useful in postoperative intractable chylothorax after surgery for esophageal cancer: a case report. J Nippon Med Sch.

[16] Liu L, Gong L, Zhang M, Wu W (2021). The effect of prophylactic thoracic duct ligation during esophagectomy on the incidence of chylothorax and survival of the patients: an updated review. Postgrad Med.

